# Prevalence, risk factors and co-morbidities of diabetes among adults in rural Saskatchewan: the influence of farm residence and agriculture-related exposures

**DOI:** 10.1186/1471-2458-13-7

**Published:** 2013-01-05

**Authors:** Roland Dyck, Chandima Karunanayake, Punam Pahwa, Louise Hagel, Josh Lawson, Donna Rennie, James Dosman

**Affiliations:** 1 Department of Medicine, College of Medicine, University of Saskatchewan, Royal University Hospital, 103 Hospital Drive, Saskatoon, Saskatchewan, S7N 0W0, Canada; 2 Department of Community Health and Epidemiology, College of Medicine, University of Saskatchewan, Saskatoon, Canada; 3 College of Nursing, University of Saskatchewan, Saskatoon, Canada; 4 Canadian Centre for Health and Safety in Agriculture, College of Medicine, University of Saskatchewan, Saskatoon, Canada

**Keywords:** Diabetes, Rural, Agriculture, Insecticides, Farm, Exposures

## Abstract

**Background:**

Although rural Canadians are reported to have higher rates of diabetes than others, little is known about the relative influence of known versus agriculture-related risk factors. The purpose of this research was to carry out a comprehensive study of prevalence, risk factors and co-morbidities of diabetes among adults in rural Saskatchewan and to determine possible differences between those living on and off farms.

**Methods:**

In 2010, we conducted a baseline mail-out survey (Saskatchewan Rural Health Study) of 11,982 households located in the province^′^s four agricultural quadrants. In addition to self-reported physician-diagnosed diabetes, the questionnaire collected information from farm and small town cohorts on possible diabetes determinants including lifestyle, family history, early life factors and environmental/agricultural-related exposures. Clustering effect within households was adjusted using Generalized Estimating Equations approach.

**Results:**

Responses were obtained from 4624 (42%) households comprising 8208 males and females aged 18 years or older and 7847 self-described Caucasian participants (7708 with complete information). The overall age-standardized diabetes prevalence for the latter was 6.35% but people whose primary residence was on farms had significantly lower diabetes prevalence than those living in non-farm locations (5.11% versus 7.33% respectively; p<0.0001). Diabetes risk increased with age and affected almost 17% of those older than 65 (OR 2.57; CI^′^ 1.63, 4.04 compared to those aged 18–45). Other known independent risk factors included family history of diabetes (OR 2.50 [CI^′^s 1.94, 3.23] if father; OR 3.11 [CI^′^s 2.44, 3.98] if mother), obesity (OR 2.66; CI^′^s 1.86, 3.78), as well as lower socioeconomic status, minimal/no alcohol intake and smoking. The most original finding was that exposure to insecticides conferred an increased risk for diabetes among males (OR 1.83; CI^′^s 1.15, 2.91). Finally, the co-morbidities with the strongest independent association with diabetes were heart disease and hypertension.

**Conclusions:**

While known diabetes risk factors are important determinants of diabetes in the agricultural zones of Saskatchewan, on-farm residence is protective and appears related to increased outdoor activities. In contrast, we have now shown for the first time that exposure to insecticides is an independent risk factor for diabetes among men in rural Canada.

## Introduction

Over the past several decades, type 2 diabetes mellitus has emerged as one of the most important chronic diseases affecting Canadian adults [1], largely related to changing lifestyles and other environmental factors associated with increasing rates of overweight/obesity [2]. Diabetes now accounts for a substantial proportion of the morbidity caused by blindness, lower limb amputations and end stage renal failure as well as deaths caused by coronary artery disease and stroke [3]. These chronic complications not only have an enormous impact on affected individuals and their families but also consume an increasing share of health care resources [3]. Despite these sobering facts, quality of diabetes care as reflected by achievement of clinical practice guidelines is less than optimal [4] and effective primary prevention initiatives remain elusive.

In Saskatchewan, we recently reported on the epidemiology of diabetes among First Nations and non-First Nations adults from 1980 to 2005 using health care system administrative databases [5]. During that period, diabetes prevalence more than doubled among both First Nations and non-First Nations women and more than tripled among their male counterparts. By 2005, 6.24% of non-First Nations men and 5.51% of non-FN women had diabetes while age adjusted diabetes prevalence was 16.01% and 20.33% among First Nations men and women respectively. Unfortunately, because of the limitations of administrative data, we were not able to study the contribution of known diabetes risk factors. We were also not able to determine if there were differences in rates of diabetes between urban and rural dwellers in Saskatchewan.

Given the impact of diabetes at the individual and social level as well as the lack of research regarding diabetes in rural populations, the purpose of this research was to use data from the Saskatchewan Rural Health Study [6] to carry out a comprehensive study of prevalence, risk factors and co-morbid conditions among a sample of non-First Nations adults living in the agricultural zones of Saskatchewan. By doing so, we sought to determine if there were differences in diabetes prevalence between rural dwellers and its known occurrence in the overall population of Saskatchewan [5], whether diabetes rates varied by agricultural region and farm/non-farm habitation, and if there were unique risk factors for diabetes that were related to agricultural practices.

## Methods

### Study design and population

The Saskatchewan Rural Health Study (SRHS) is a prospective cohort study examining the health of people living in rural Saskatchewan and includes both a baseline phase in 2010 (detailed descriptions of survey and variables reported elsewhere) [6] and a follow-up phase planned for 2014. The baseline survey phase of the study was approved by the University of Saskatchewan biomedical ethics review board. Briefly, 39 of the 297 rural municipalities and 16 of the 145 towns (usual population 500 to 5000) in Saskatchewan were selected to participate in the study. These rural municipalities and towns were selected at random from four quadrants of the province (Southeast, Southwest, Northeast, and Northwest). The local councils for 32 (82%) of 39 rural municipalities and 15 (94%) of 16 towns agreed to participate on behalf of their residents and supplied mailing addresses for the survey. Dillman^′s^ method, which involves a series of mail contacts with all prospective participants, was utilized to recruit study participants [6,7]. After excluding ineligible households (e.g. addresses unknown or outside study area, duplicates, deceased), information on variables based on the Population Health Framework [8,9] and described below was collected by self-administered questionnaires. These questionnaires were mailed to 11,004 households. Some measures of lifestyle factors, occupational exposures, and socio-economic status used in our questionnaire were adopted from previous research studies that had validated these measures [8,10,11].

### Primary health outcome

The primary outcome for this part of the study was self-reported physician-diagnosed diabetes, as determined from the baseline survey question: “Has a doctor ever said you had … diabetes”.

### Contextual factors

The contextual factors of interest in the study were: (i) rural dwelling – residence on a farm or non-farm location (including town and self-described acreage), (ii) socioeconomic status (household income adequacy) – household income adequacy was a derived variable with four categories based on total household income and number of people living in the household according to the Statistics Canada definition [12], and (iii) interior environment (smoking inside the house) – based on whether or not any household dweller used cigarettes, cigars and/or pipes in the home.

### Individual factors

The individual factors considered were: (i) family history of diabetes among first degree relatives (father, mother, brother/sister), (ii) lifestyle or behavior-related factors including smoking, alcohol use, body mass index (BMI) calculated by dividing weight in kg by height in m^2^ (overweight =BMI 25–29.9; obesity = BMI 30 and higher),physical activity and television and computer viewing time; (iii) environmental and occupational exposures including grain dust, mine dust, asbestos dust, wood dust, other dust, livestock, smoke from stubble burning, diesel fumes, welding fumes, solvent fumes, oil/gas well fumes, herbicides, fungicides, insecticides, molds, radiation and other exposures (information was also collected on the frequency and duration of exposures) and (iv) individual educational attainment.

### Covariates

Information was obtained on important covariates such as age, sex, marital status, ethnicity and selected co-morbid conditions including hypertension, heart disease, heart attack, stroke, hardening of the arteries, tuberculosis, cancer and several indicators of chronic lung disease.

### Statistical analysis

Statistical analysis was conducted using SAS 9.02 (SAS Institution, Cary, NC). Prevalence was presented as observed/total and percentage. We calculated both crude and age adjusted prevalence standardized to the 2006 Canadian census population. We also calculated age adjusted prevalence standardized to the 1991 Canadian census population for participants aged 20 and older so that comparisons to historical data could be made. Chi-square tests were used to determine the univariate association of prevalence of diabetes and location of residence. Logistic regression models were used to predict the relationship between a binary diagnosis of diabetes (yes or no) and a set of explanatory variables. A multilevel logistic regression modeling approach based on a generalized estimating equations, with individuals (1st level) nested within households (2nd level), was utilized to evaluate the effects of both contextual and individual factors after adjustment for covariates of interest. This accounts for the within-subject dependencies that occur in the analysis due to multiple people from the same household. A series of multi-level models were fitted to determine whether potential risk factors, confounders, and interactive effects (e.g. individual and contextual risk factors) contributed significantly to the prevalence of diabetes. Based on bi-variable analysis, variables with p<0.20 were candidates for the multivariate model. All variables that were statistically significant (p<0.05) as well as important contextual factors (location of residence), were retained in the final multivariable model. A parsimonious model was selected based on QIC (Quasi likelihood under the Independence model Criteria) goodness-of-fit statistic [13,14]. The strength of associations is presented by odds ratios (OR) and their 95% confidence intervals (CI).

## Results

Table 1 shows baseline information for the study population in 2010. Of the 11,004 eligible addresses in the study areas to which surveys were sent, responses were obtained from 4624 (42%) households comprising 8208 males and females aged 18 years or older who lived on an identified farm/non-farm location. Because very few individuals indicated ethnic background as First Nations, Metis or other (or the question was not answered), we subsequently confined our analyses to the 7708 people with a self-described Caucasian heritage. Table 1 also shows distribution of study participants and crude diabetes prevalence by geographic location and farm/non-farm residence. Although overall unadjusted diabetes prevalence was similar between Saskatchewan^′^s agricultural zones, it ranged from 8.0% (lowest) in the northwest quadrant to 10.4% (highest) in the northeast quadrant and, in each quadrant, was significantly higher among non-farm compared to farm residents. Overall, 10.7% of non-farm residents and 6.9% of farm residents reported a physician-diagnosis of diabetes (p<0.001). Table 2 shows the overall crude as well as age-standardized prevalence of diabetes by residence location in those aged 20 and older. After adjusting for age, diabetes prevalence remained significantly higher for those living in non-farm compared to farm locations (7.3% and 5.1% respectively; p<0.0001).

**Table 1:** **Saskatchewan rural health study** – **study populations and diabetes numbers** – **adults 18 and older**

	**FARM**	**NON**-**FARM**	**TOTAL**
Eligible Household Addresses	---	---	11004
Household Responses (Rate %)	---	---	4624 (42.0)
Persons Participating	3445	4763	8208
Mean Age (+/-standard error)	55.0 (0.24)	56.8 (0.24)	56.1 (0.17)
Males: Females (Ratio)	1794/1650 (1.09)	2246/2514 (0.89)	4040/4164 (0.97)
Caucasian Heritage & Diabetes Information	3296	4412	7708
Total self-reported diabetes (crude %)	227 (6.9)***	472 (10.7)***	699 (9.1)
Quadrant of SK - #diabetes/total (%)			
Southwest	34/528 (6.4)*	93/923 (10.1)*	127/1451 (8.7)
Southeast	41/671 (6.1)**	109/998 (10.9)**	150/1669 (9.0)
Northwest	58/966 (6.0)**	132/1401 (9.4)**	190/2367 (8.0)
Northeast	94/1131 (8.3)**	138/1090 (12.7)**	232/2221 (10.4)

*p<0.05.

**p<0.01.

***p<0.001.

**Table 2:** **Crude** &**age standardized diabetes prevalence by farm**/**non**-**farm residence** –**adults 18 and older**

	**DIABETES CASES**	**CRUDE PREVALENCE**	**AGE**-STANDARDIZED*				
Total	699/7708	9.07	6.35	(5.79)
Farm	227/3296	6.89	5.11	(4.67)
Non-Farm	472/4412	10.69	7.33**	(6.67)**

Standardized to 2006 Canadian Census population.

* Values in brackets are standardized to 1991 Canadian census & include participants aged 20 and older (see methods).

**p<0.0001.

Crude diabetes prevalence was significantly higher (P=0.002) among men (10.1%) compared to women (8.1%). However, when differences in age between males and females were taken into account, there was no significant difference in diabetes prevalence at 6.5% and 6.1% respectively.

Tables 3 and 4 show the unadjusted relationships between key variables and diabetes. Those with diabetes were more likely to be older, married or widowed, and to have lower levels of income and education. Diabetes was also more common among those living in non-farm dwellings and those residing in the north-east agricultural zone of Saskatchewan. However, for those living on farms, diabetes risk was not related to the type of agricultural activity (growing grain versus raising cattle). Hereditary and early life factors clearly discriminated between those with and without diabetes. The impact of family history was particularly striking with progressively increasing diabetes risk with a diabetic father, diabetic mother and both parents having diabetes. Diabetes was also more likely among those who had lower birth weights (<2500 grams), had been breast fed and had lived on a farm during their first year of life. Maternal smoking status during pregnancy did not affect diabetes risk.

**Table 3:** Relationship between diabetes and individual characteristics on univariate analysis

**PARAMETER**	**DIABETES PREVALENCE(%)**	**ODDS RATIO(95% Confidence intervals)**	**PARAMETER**	**DIABETES PREVALENCE(%)**	**ODDS RATIO(95% Confidence intervals)**
Sex			Location of Home		
Male	10.0	1.27 (1.10, 1.48)	Farm	6.9	0.62 (0.52, 0.73)
Female	8.1	1.00 (ref)	Non-Farm	10.7	1.00 (ref)
Age			Family History		
18-45 years	2.4	1.00 (ref)	Dad diabetic		
46-55 years	6.3	2.75 (1.93, 3.92)	Yes	16.0	2.42 (1.99, 2.93)
56-65 years	10.7	4.85 (3.46, 6.80)	No	7.2	1.00 (ref)
>65 years	15.7	7.52 (5.44, 10.39)	Mom diabetic		
Marital Status			Yes	20.1	3.52 (2.94, 4.21)
Married	9.2	1.55 (1.07, 2.23)	No	6.6	1.00 (ref)
Common law	5.6	0.90 (0.49, 1.64)	Both diabetic		
Widowed	12.6	2.19 (1.42, 3.40)	Yes	29.5	4.80 (3.41, 6.77)
Divorced/separated	9.8	1.66 (0.97, 2.87)	No	7.9	1.00 (ref)
Never married	6.1	1.00 (ref)	Farm Age 0-1		
Education Attained			Yes	9.5	1.19 (1.00, 1.41)
<High school	14.4	2.24 (1.81, 2.77)	No	8.0	1.00 (ref)
High school	8.4	1.24 (1.00, 1.54)	Mom Smoked		
University	4.9	0.71 (0.50, 1.01)	Yes (pregnancy)	8.4	0.94 (0.75, 1.18)
Other post-2nd	6.9	1.00 (ref)	Do not know	10.4	1.16 (0.92, 1.48)
Income Adequacy [6]			No (pregnancy)	9.0	1.00 (ref)
Lowest	17.2	3.26 (2.06, 4.70)	Birth Weight		
Lower middle	13.2	2.37 (1.87, 2.99)	Don’t know	10.7	1.55 (1.30, 1.85)
Upper middle	10.3	1.79 (1.45, 2.20)	<2500 g	10.7	1.57 (1.11, 2.20)
Highest	6.0	1.00 (ref)	2500-3999 g	7.1	1.00 (ref)
Quadrant (Region)			>= 4000 g	7.3	1.04 (0.73, 1.48)
Southwest	8.7	1.09 (0.86, 1.39)	Breastfed		
Southeast	8.9	1.13 (0.90, 1.43)	Yes	9.1	1.31 (1.07, 1.60)
Northeast	10.4	1.33 (1.08, 1.64)	Don’t know	10.8	1.56 (1.26, 1.94)
Northwest	8.0	1.00 (ref)	No	7.1	1.00 (ref)

**Table 4:** Relationship between diabetes and individual characteristics on univariate analysis

**PARAMETER**	**DIABETES PREVALENCE(%)**	**ODDS RATIO(95% Confidence intervals)**	**PARAMETER**	**DIABETES PREVALENCE (%)**	**ODDS RATIO(95% Confidence intervals)**
**LIFE STYLE**			TV Video/Week		
Body Mass Index			None	6.6	1.00 (ref)
Normal	4.4	1.00 (ref)	< 1 hour	12.4	1.98 (0.87, 4.52)
Overweight	7.3	1.72 (1.35, 2.18)	1-2 hours	8.2	1.20 (0.57, 2.54)
Obese	16.1	4.13 (3.29, 5.18)	3-5 hours	8.7	1.30 (0.64, 2.64)
Exercise (per day)			6-10 hours	7.5	1.13 (0.55, 2.30)
None	13.2	2.36 (1.52, 3.67)	11-14 hours	7.8	1.17 (0.56, 2.41)
< 15 minutes	14.1	3.14 (1.88, 5.23)	15-20 hours	8.7	1.30 (0.64, 2.67)
15-30 minutes	9.2	1.98 (1.25, 3.12)	> 20 hours	13.6	2.16 (1.06, 4.40)
31-60 minutes	5.0	1.05 (0.64, 1.72)	**EXPOSURES**		
> 60 minutes	4.8	1.00 (ref)	Livestock		
Smoking Status			Yes	9.7	1.25 (1.07, 1.46)
Current smoker	8.3	1.21 (0.93, 1.58)	No	8.0	1.00 (ref)
Ex-smoker	12.3	1.84 (1.56, 2.17)	Stubble Burn		
Never smoker	7.0	1.00 (ref)	Yes	9.9	1.25 (1.06, 1.46)
Alcohol Intake			No	8.1	1.00 (ref)
Never	14.0	1.00 (ref)	Herbicides		
Up to one/month	11.1	0.77 (0.63, 0.95)	Yes	9.4	1.17 (0.99, 1.36)
2-4 times/month	6.3	0.41 (0.32, 0.52)	No	8.3	1.00 (ref)
2-3 times /week	4.3	0.28 (0.20, 0.39)	Fungicides		
4-7 times /week	8.4	0.57 (0.43, 0.77)	Yes	9.9	1.21 (1.03, 1.43)
Computer Use/Week			No	8.3	1.00 (ref)
None	13.7	1.00 (ref)	Insecticides		
< 1 hour	7.4	0.51 (0.38, 0.67)	Yes	9.6	1.19 (1.02, 1.40)
1-2 hours	6.3	0.43 (0.34, 0.56)	No	8.2	1.00 (ref)
3-5 hours	8.1	0.56 (0.44, 0.71)	Other Dust		
6-10 hours	5.0	0.34 (0.25, 0.47)	Yes	7.9	0.84 (0.70, 1.01)
11-14 hours	7.2	0.50 (0.33, 0.75)	No	9.3	1.00 (ref)
15-20 hours	6.8	0.46 (0.28, 0.76)			
>20 hours	9.3	0.66 (0.45, 0.96)			

Several measures reflecting lifestyle were significantly associated with diabetes including increasing body mass index, decreasing daily time spent at exercise and being a current or ex-smoker. Alcohol consumption appeared protective as did increasing time spent at a computer. In contrast, watching television or videos was significantly associated with diabetes but only when it occupied >20 hours per week.

For the most part, agricultural and other rural exposures were not significantly associated with diabetes. These included grain, mine, asbestos and wood dusts; molds; diesel, welding, solvent and oil/gas well fumes; and radiation exposure. However, Table 4 shows that exposure to livestock, smoke from stubble burning, and herbicides, fungicides and insecticides resulted in modest but significant relationships with diabetes. Exposure to “other” dusts (mostly from households, soil/dirt, roads, fertilizers, construction and forage crops) was inversely related to diabetes.

Table 5 summarizes the physician-diagnosed co-morbidities associated with diabetes. Cardiovascular disorders exhibited odds ratios (ORs) of 3.01 (hardening of the arteries) to 5.21 (high blood pressure) among people with diabetes compared to others, while tuberculosis also demonstrated a strong relationship. Cancer and chronic lung disorders displayed less striking but still largely significant associations with diabetes. Diagnosed sleep apnea and related symptoms (Epworth sleepiness scale and snoring) demonstrated particularly significant associations with diabetes.

**Table 5:** **Prevalence of diabetes and univariate associations with co**-**morbidities**

**PARAMETER**	**DIABETES PREVALENCE(%)**	**ODDS RATIO(95% Confidence intervals)**	**PARAMETER**	**DIABETES PREVALENCE(%)**	**ODDS RATIO(95% Confidence intervals)**
**DIAGNOSED**:			Emphysema		
Heart Disease			Yes	11.4	1.22 (0.67, 2.24)
Yes	21.7	3.15 (2.52, 3.93)	No	8.9	1.00 (ref)
No	7.9	1.00 (ref)	COPD		
Heart Attack			Yes	17.1	2.09 (1.39, 3.14)
Yes	21.9	3.00 (2.27, 3.96)	No	8.7	1.00 (ref)
No	8.4	1.00 (ref)	Asthma		
Hardened Arteries			Yes	10.3	1.16 (0.91, 1.49)
Yes	21.7	3.01 (2.20, 4.11)	No	8.9	1.00 (ref)
No	8.3	1.00 (ref)	Sleep Apnea		
High Blood Pressure			Yes	16.6	2.13 (1.64, 2.76)
Yes	18.7	5.21 (4.40, 6.17)	No	8.4	1.00 (ref)
No	4.2	1.00 (ref)	**OTHER**:		
Stroke			Sleepiness Scale*		
Yes	26.3	3.73 (2.59, 5.35)	Abnormal	12.0	1.52 (1.25, 1.86)
No	8.6	1.00 (ref)	Normal	8.1	1.00 (ref)
Tuberculosis			Do You Snore		
Yes	25.8	3.47 (1.51, 7.96)	Yes	10.3	2.09 (1.70, 2.58)
No	8.9	1.00 (ref)	No	5.2	1.00 (ref)
Cancer			General Health**		
Yes	12.4	1.45 (1.12, 1.86)	Excellent	0.7	1.00 (ref)
No	8.7	1.00 (ref)	Very Good	3.3	4.6 (1.9, 11.4)
Chronic Bronchitis			Good	10.8	16.5 (6.8, 39.8)
Yes	12.0	1.41 (1.04, 1.89)	Fair	22.2	38.8 (16.0, 94.1)
No	8.7	1.00 (ref)	Poor	27.4	51.0 (20.1, 129.5)

*Epworth Sleepiness Scale

**Self-Reported.

Table 6 provides the results of the final logistic regression model of the relationship between independent variables and self-reported physician-diagnosed diabetes. After adjusting for other variables in the model, sex, marital status, education level and agricultural quadrant of Saskatchewan were no longer significantly associated with diabetes. However, decreasing income and non-farm rural dwelling remained important independent predictors of diabetes as did family history of diabetes. With respect to life style factors, being obese continued to be a significant risk factor for diabetes while regular alcohol consumption remained protective. Exposure to dusts other than from grain, mine, asbestos and wood also appeared to protect against diabetes.

**Table 6:** Odds ratios of diabetes risk based on multivariate logistic regression analysis

**PARAMETER**	**ODDS RATIO(95% Confidence intervals)**	**SIG P Value**	**PARAMETER**	**ODDS RATIO(95% Confidence intervals**)	**SIG P value**
**PERSONAL**:			**LIFE STYLE**:		
Sex: Male	0.70 (0.45, 1.08)	0.1103	Body Mass Index		
Female	1.00 (ref)		Normal	1.00 (ref)	
Age: 18-45 years	1.00 (ref)		Overweight	1.27 (0.88, 1.82)	0.2047
46-55 years	1.68 (1.10, 2.56)	0.016	Obese	2.66 (1.86, 3.78)	<0.0001
56-65 years	2.27 (1.49, 3.45)	0.0001	Smoking Status		
>65 years	2.57 (1.63, 4.04)	<0.0001	Current Smoker	0.90 (0.47, 1.73)	0.7586
Income Adequacy			Ex-smoker	1.01 (0.69, 1.48)	0.954
Lowest	1.95 (1.14, 3.36)	0.0151	Never Smoker	1.00 (ref)	
Lower Middle	1.57 (1.10, 2.23)	0.0134	Alcohol Intake		
Upper Middle	1.47 (1.12, 1.94)	0.0059	Never	1.00 (ref)	
Highest	1.00 (ref)		Up to one/month	1.05 (0.77, 1.44)	0.7328
Quadrant (region)			2-4 times/month	0.62 (0.44, 0.88)	0.0081
Southwest	1.11 (0.80, 1.54)	0.5469	2-3 times/week	0.38 (0.23, 0.63)	0.0001
Southeast	0.85 (0.61, 1.18)	0.3272	4-7 times/week	0.65 (0.42, 1.00)	0.0495
Northeast	1.00 (0.75, 1.34)	0.9816	**CO**-**MORBITIES**:		
Northwest	1.00 (ref)		Heart Disease		
Location of Home			Yes	1.56 (1.12, 2.19)	0.0094
Farm	0.71 (0.56, 0.92)	0.0082	No	1.00 (ref)	
Non-farm	1.00 (ref)		High BP		
**DIABETES**:			Yes	3.23 (2.53, 4.13)	<0.0001
Dad: Yes	2.50 (1.94, 3.23)	<0.0001	No	1.00 (ref)	
No	1.00		Do You Snore		
Mom: Yes	3.11 (2.44, 3.98)	<0.0001	Yes	1.63 (1.19, 2.23)	0.0022
No	1.00 (ref)		No	1.00 (ref)	
**EXPOSURES**:			**INTERACTIONS**:		
Other Dusts			Sex & Smoking		
Yes	0.75 (0.59, 0.96)	0.0246	Male* Smoker	2.68 (1.22, 5.90)	0.0142
No	1.00 (ref)		Male* Ex-smoker	1.98 (1.19, 3.30)	0.009
Insecticides			Sex & Insecticides		
Yes	0.81 (0.56, 1.17)	0.2610	Male* Insecticides	1.83 (1.15, 2.91)	0.0113
No	1.00 (ref)				

**Figure 1:**
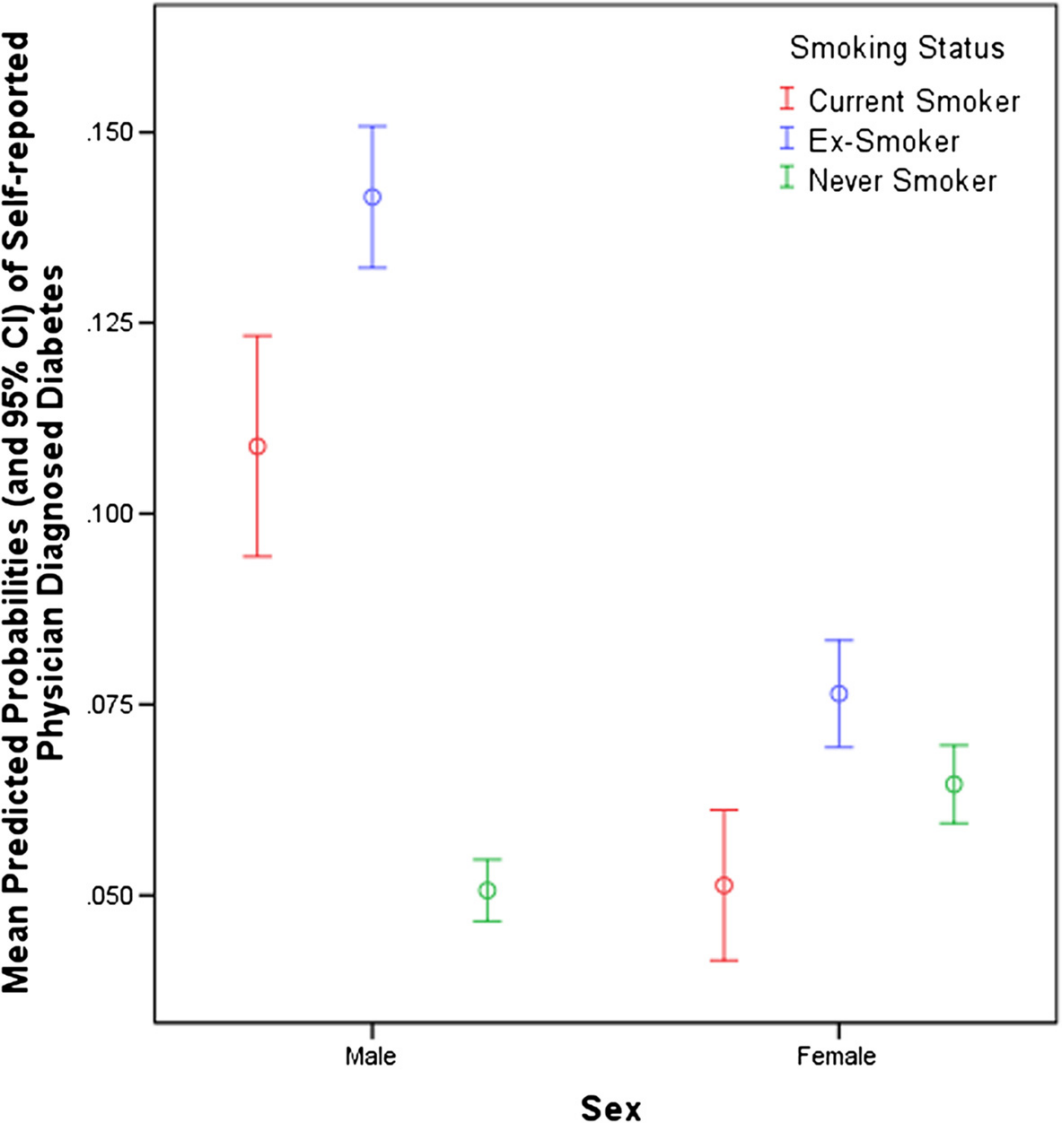
Interaction between Smoking Status and Sex.

**Figure 2:**
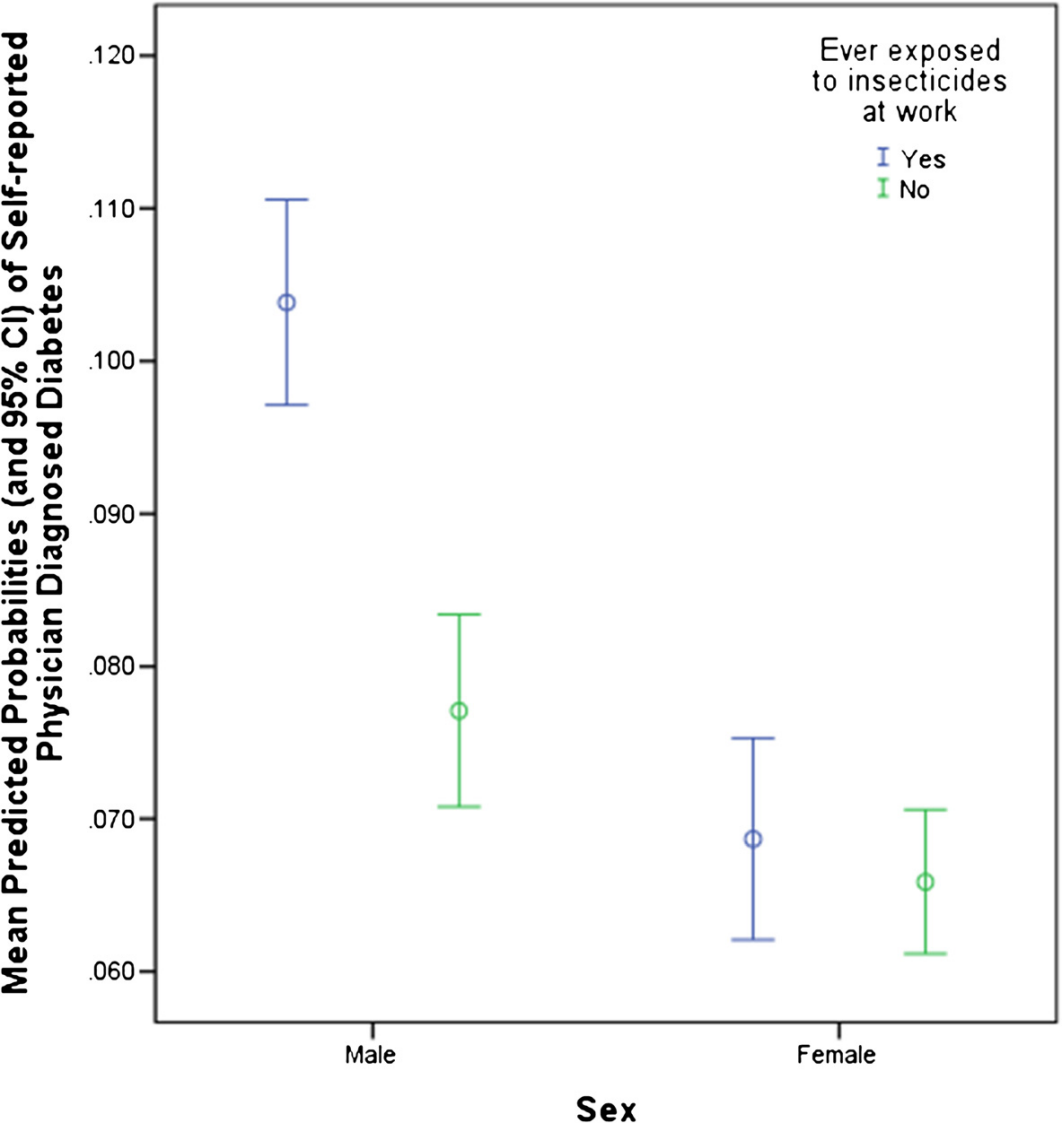
Interaction between Insecticide Exposure and Sex.

Figures 1 and 2 show that both smoking and exposure to insecticides were significant predictors of diabetes in an interaction with male sex. Current male smokers were almost three times more likely and male ex-smokers almost twice as likely as male non-smokers to have diabetes while diabetes risk for women was not significantly associated with smoking status. Similarly, insecticide exposure almost doubled the risk for diabetes among men but was not a predictor of diabetes among women. In a sub-analysis of the interaction between sex and insecticides that determined diabetes prevalence for men by age group (not shown), there was a trend for higher diabetes prevalence in younger men (age groups ≤65 years) exposed to insecticides compared to those >65% years.

With respect to co-morbidities, only heart disease, high blood pressure and snoring were independently related to diabetes when the general health index was excluded from the model.

## Discussion

This study describes the epidemiology of diabetes among adults of self-identified Caucasian heritage living in the agricultural area of rural Saskatchewan in 2010. Although the overall diabetes prevalence of 6.4% age standardized to the 2006 Canadian population was slightly higher than the combined prevalence (5.9%) for rural plus urban non-First Nations males and females in Saskatchewan in 2005 [5], when we standardized this study^′^s data to the same Canadian census population (1991) as the latter study, the value of 5.8% was almost identical. We did not observe the higher prevalence of diabetes among males in rural Saskatchewan as we had for the province overall [5] but did find that people whose primary residence was on farms had significantly lower diabetes prevalence than those living in non-farm locations. As with other Canadians [1], diabetes risk increased with age and affected almost 17% of those older than 65. Other known independent risk factors included family history of diabetes, obesity, lower socioeconomic status, minimal/no alcohol intake and smoking. The most original finding of this study was that exposure to insecticides conferred an increased risk for diabetes among males. Finally, the co-morbidities with the strongest independent association with diabetes among rural adults in Saskatchewan were heart disease and hypertension.

Although rural Canadians [15] including those in Saskatchewan [16] are reported to have higher diabetes prevalence than those living in urban centers, our findings suggest that this may only be true for people living in non-farm locations. Diabetes prevalence in the latter was 7.3% compared to 5.1% among those living on farms. After adjusting for possible differences in age, obesity levels and other known diabetes risk factors, it is likely that there are at least two reasons for this observation. First, many people who retire from farming activities move into local communities and some do so because of pre-existing health conditions including diabetes. Second, despite increased mechanization, farming still requires being physically active and working outside. These activities are protective against diabetes [17,18] and may explain the finding that exposure to “other dusts” was associated with a lower risk of diabetes. In fact, “other dusts” largely included those encountered outdoors and we believe it is a surrogate for time spent outside – this is more likely among physically active people including farmers.

As expected, known risk factors for diabetes were significant independent diabetes predictors in this study as well. The exception was evidence of physical inactivity (minimal daily exercise duration and prolonged television viewing) that were associated with diabetes on univariate analysis but not after adjusting for other variables. Interestingly, having a mother with diabetes was a stronger determinant of diabetes than having a diabetic father and may relate to the increased intergenerational risk for diabetes conferred by diabetic pregnancies [19]. As others have shown [20], we found a protective effect of moderate alcohol consumption on diabetes risk compared to minimal intake. Heavier alcohol intake (4–7 times per week) was also protective but not to the same degree. We also found that smoking was a risk factor for diabetes but only among men. Large studies have shown a link between smoking and type 2 diabetes in both men and women [21] but this association is dose-related [22] and the interaction that we found with sex could possibly be explained by heavier smoking among men.

Having diabetes was strikingly related to study participants^′^ perception of declining general health. Not surprisingly, multivariate analysis showed that heart disease and hypertension were the most significantly associated co-morbidities but snoring was also strongly and independently linked to diabetes. The latter is most likely due to the known relationship between type 2 diabetes and obstructive sleep apnea [23]. This relationship is due at least in part to their common risk factor of obesity although there may be other interrelated factors that contribute to the pathogenesis of both.

As far as we are aware, this is the first study to show a relationship between insecticide exposure and diabetes among Canadian farmers and one of few studies to observe this relationship after adjusting for the contribution of both a large number of other environmental exposures and known diabetes risk factors. There is increasing evidence for a cause-effect link between environmental toxicants and diabetes [24-27] that is related to adipose tissue accumulation and reported effects on insulin production/resistance [28,29]. As with smoking, we found an interaction with sex; men but not women experienced a significant risk of diabetes with insecticide exposure and a plausible explanation relates to the fact that men are more likely to work directly with these products. Importantly, we also found that this relationship has been stronger in recent decades than previously. This may indicate that, despite better equipment and safer use of chemicals, our increasing dependency on their use to support modern agricultural practices is not without significant health risks. Interestingly, although herbicides and fungicides also demonstrated positive associations with diabetes in our univariate analysis, they did not emerge as independent risk factors in multivariate analysis. However, it is likely that there is substantial overlap in the use of all three products, so we are unable to determine if and by how much these chemicals interacted in elevating diabetes risk.

Strengths of this study included its large number of participants, the widely diverse areas of Saskatchewan from which the study population was drawn, stratification by farm/non-farm habitation and our ability to evaluate the relative contribution of an extensive number of both known and potential diabetes risk factors related to personal as well as agricultural activities. Although we were not able to evaluate the role of ethnicity in diabetes risk, we have to some extent controlled for possible genetic factors in our analyses by only including people of European heritage. Limitations included the cross sectional nature of the study and the associated inability to be confident about cause-effect relationships. We were not able to distinguish between types 1 and 2 diabetes but over 90% of diabetic adults have type 2 diabetes. Although there was a relatively large proportion of non-participation, the moderate response rate of 42% is consistent with mail-out surveys without inducement. Our sample had a similar gender distribution, but a larger proportion of older people, compared to the overall Saskatchewan population residing outside of cities and First Nations reserves. Therefore we may not be able to generalize our findings to the total provincial rural population. There were possible inaccuracies related to self-reporting of both diabetes and potential diabetes risk factors. For example, we were not able to determine the degree of exposure to possible toxicants or their chemical class. Nonetheless, the fact that the relationship between insecticides and diabetes has been shown in other studies, and that we demonstrated an interaction with sex when males are known to be more engaged in chemical spraying, strengthens the biologic plausibility of our findings. Finally, in univariate analysis, we found that maternal breast feeding was predictive of diabetes in the offspring and maternal smoking did not influence the risk for diabetes in the offspring. These somewhat unexpected findings may have been due to a large proportion of respondents not knowing whether or not their mothers had engaged in these practices and an attendant inability to obtain accurate information.

## Conclusions

This is the most comprehensive study yet published of diabetes among non-Aboriginal people living in a large rural and predominantly agricultural area of Canada. While previous reports found a higher prevalence of diabetes in rural Canadians, our research found that this was only true for people living in non-farm locations and that those living on farms actually had lower age standardized diabetes rates. Indirect evidence suggests that this may be partly due to increased outdoor activities. In addition to known diabetes risk factors, exposure to insecticides was an independent predictor for diabetes among men. This new finding requires corroboration as well as further studies to define the degree of risk associated with specific agents. However, it is a sobering reminder that modern agricultural practices not only carry with them immediate threats from injuries and noxious exposures, but can also lead to long term health consequences. On an individual level, educational programs aimed at safe use of chemicals and spraying equipment should include awareness of this possible link. On a population level, this information should be part of the debate around the use and impact of such agents on the health of humans, other species and our environment.

## Competing interests

The authors declare that they have no competing interests.

## Authors’ contributions

RD contributed to the design of the study, researched data, contributed to the discussion and wrote the manuscript. CK and PP contributed to the design of the study, performed the statistical analysis, researched data, contributed to the discussion and reviewed/edited the manuscript. LH, JL, DR and JD helped to design the study, contributed to the discussion and reviewed/edited the manuscript. All authors read and approved the final manuscript.

## Pre-publication history

The pre-publication history for this paper can be accessed here:

http://www.biomedcentral.com/1471-2458/13/7/prepub
